# A novel peanut allergoid is safe and effective in immunotherapy in a peanut allergy mouse model

**DOI:** 10.1186/2045-7022-3-S3-P1

**Published:** 2013-07-25

**Authors:** J Smit, R Pieters, M van Roest, L Kruijssen, S Koppelman, D-J Opstelten, H Van der Kleij

**Affiliations:** 1 Immunotoxicology, Institute for Risk Assessment Sciences, Utrecht, the Netherlands; 2 HAL Allergy BV, Leiden, the Netherlands

## Background

Peanut is one of the most common foods responsible for food-induced anaphylaxis in adults. Unfortunately, commonly used allergen-specific immunotherapy has not been successful for the treatment of food allergy because of the high risk of serious side-effects. Therefore, chemically modified allergen extracts with improved safety characteristics are being investigated for its potential use in immunotherapy.

## Methods

Peanut extract (PE) from de-fatted peanut powder was modified by reduction of disulfide bonds and subsequent alkylation of the free Cys residues resulting in an allergoid PE (mPE). The potency of PE and mPE to induce PE-specific IgG was evaluated after i.p. injections in mice. Subsequently, mice were sensitized intra-gastrically for PE and either 1) subcutaneously challenged with different concentrations of PE or mPE to assess the safety profile of these product candidates, or 2) de-sensitized with subcutaneous injections of either PE or mPE (immunotherapy) for 4-6 weeks, followed by oral and i.p. challenges to assess the efficacy profile of the preparations. To assess the safety and efficacy profile of mPE compared to PE, body temperature was measured after challenge as an objective parameter of an anaphylactic shock response. In addition, during the course immunotherapy, blood samples were taken for analysis of antibody responses and mast cell activation.

## Results

PE and mPE were equally potent in inducing PE-specific IgG antibodies in mice. Mice sensitized for PE experienced severe anaphylactic symptoms upon subcutaneous challenge with PE. Modified PE did not give rise to such reactions, even when given up to 100 fold higher dosages. Immunotherapy with both PE and mPE resulted in a significant reduction of the anaphylactic shock response upon systemic challenge. In addition, both PE and mPE were able to induce strong increases in the levels of PE-specific IgG1 and IgG2a compared to non-desensitized mice. Surprisingly, the mucosal mast cell response after challenge was decreased after immunotherapy with PE but not with mPE.

## Conclusion

Using *in vivo* mouse models, we have shown that an allergoid preparation of peanut extract has a significantly improved safety profile compared to its native counterpart while retaining its immunogenicity and efficacy profile. Furthermore, this study supports the usefulness of mouse models in testing safety, immunogenicity and efficacy of new immunotherapeutic preparations.

## Disclosure of interest

None declared.

